# Circulatory miR-133b and miR-21 as Novel Biomarkers in Early Prediction and Diagnosis of Coronary Artery Disease

**DOI:** 10.3390/genes11020164

**Published:** 2020-02-05

**Authors:** Dinesh Kumar, Rajiv Narang, Vishnubhatla Sreenivas, Vandana Rastogi, Jagriti Bhatia, Daman Saluja, Kamna Srivastava

**Affiliations:** 1 Dr. B R Ambedkar Center for Biomedical Research, University of Delhi, New Delhi 110007, India; dkmite@gmail.com (D.K.); dsalujach59@gmail.com (D.S.); 2 Department of Cardiology, All India Institute of Medical Sciences, New Delhi 110029, India; r_narang@yahoo.com; 3 Department of Statistics, All India Institute of Medical Sciences, New Delhi 110029, India; sreevishnubhatla30@gmail.com (V.S.); rastogi.vandana2028@gmail.com (V.R.); 4 Department of Pharmacology, All India Institute of Medical Sciences, New Delhi 110029, India; jagriti2012@rediffmail.com

**Keywords:** circulatory miRNA, coronary artery disease, miR-133b, miR-21, RT-qPCR, biomarkers

## Abstract

While coronary artery disease (CAD) has become a major threat worldwide, the timely biomarker-based early diagnosis of CAD remains a major unmet clinical challenge. We aimed towards assessing the level of circulatory microRNAs as candidates of novel biomarkers in patients with CAD. A total of 147 subjects were recruited which includes 78 subjects with angiographically proven CAD, 15 pre-atherosclerotic normal coronary artery (NCA) subjects and 54 healthy individuals. Quantitative real-time PCR assays were performed. MiR-133b was downregulated by 4.6 fold (*p* < 0.0001) whereas miR-21 was upregulated by ~2 fold (*p* < 0.0001) in plasma samples of CAD patients. Importantly, both the miRNAs showed association with disease severity as miR-133b was downregulated by 8.45 fold in acute coronary syndrome (ACS), 3.38 fold in Stable angina (SA) and 2.08 fold in NCA. MiR-21 was upregulated by 2.46 fold in ACS, 1.90 fold in SA and 1.12 fold in NCA. Moreover, miR-133b could significantly differentiate subjects with ST-elevation myocardial infarction (STEMI) from Non-STEMI. Area under the curve (AUC) for miR-133b was 0.80 with >75.6% sensitivity and specificity, AUC for miR-21 was 0.79 with >69.4% sensitivity and specificity. Our results suggest that miR-133b and miR-21 could be possible candidates of novel biomarkers in early prediction of CAD.

## 1. Introduction

Coronary artery disease has evidently become a major health burden, with life-threatening episodes representing the primary cause of morbidity and mortality in developed as well as in developing countries [1,2]. It has continued to increase worldwide at an alarming rate. CAD is characterized by coronary plaque formation and progression. Luminal narrowing due to excessive plaque buildup causes insufficient blood supply to the heart muscles and oxidative stress consequently leads to myocardial infarction (MI) [3]. Atherosclerosis, an initial physical state of CAD, is often diagnosed at very late stages, and by that time it is becoming a worse condition. It starts at young age and progresses quite silently over years from subclinical to clinical symptoms. A rapid and effective test for early prediction or detection of stable coronary disease and, more importantly, pre-atherosclerotic disease or condition is still lacking. There are certain therapeutic tests including an electrocardiogram (ECG or EKG), echocardiogram, exercise stress tests, cardiac catheterization, and biomarkers such as cardiac troponin (cTnI or cTnT), B-type natriuretic peptide (BNP), and Creatine kinase-MB (CK-MB) that lead to improved diagnosis, but are more beneficial in detecting the severe and late stage conditions such as acute myocardial infarction (AMI). Many of these biomarkers are non-specific and unreliable, and the invasive nature of these diagnostic procedures limits their application [4,5]. Thus, the regular failure of the current biomarkers in discriminating MI or various severe stages of CAD as well as the absence of any other distinguished plasma biomarker for clinical diagnosis of stable or pre-atherosclerotic condition increases the high clinical demand for the optimal management of CAD through some innovative, reliable, highly sensitive and non-invasive diagnostic biomarkers. 

MicroRNAs are an evolutionarily conserved class of small noncoding RNAs (ncRNAs) approximately ~22 nucleotides in length which have emerged as a novel class of gene regulatory molecule [6]. MicroRNAs, as endogenous molecules, have been proven to play a pivotal role in diverse area of patho-physiological processes. They provide an essential function in fundamental biological processes such as cellular development, intracellular metabolic mechanisms, cellular differentiation and proliferation, and a variety of stress responses in cardiovascular diseases, neurological disorders and various malignancies [6,7]. MicroRNAs belong to a novel class of regulators which negatively regulate gene expression at post transcriptional level and thereby affect translation of messenger RNA (mRNA) by either perfect or imperfect binding to the target mRNA counterpart within its 3’ untranslated region (3’UTR) [8]. MicroRNA expression and their profiles are highly informative in helping diagnosis and prognosis of many diseases, as well as response to therapies, and thus provide a distinctive opportunity to translate these understandings into clinical settings as miRNA-based biomarkers [9]. Identification of miRNA expression in “heart-specific” cells as well as vascular cells could lead researchers to discover important regulatory roles for miRNAs during coronary plaque formation, vessel wall inflammation and cardiac hypertrophy in adults [10,11]. This indicates their role in heart development, which is further supported by findings where defects in vessel formation, angiogenesis, and cardiac development have been observed due to depletion of ‘Dicer’, a miRNA processing enzyme [12,13].

Several studies impart information establishing miRNAs as key regulators of cardiovascular diseases [14,15]. Interestingly, few reports have proposed the diagnostic potential of miRNAs in cardiac complications such as MI [16] and heart failure [17]. A former report demonstrated that AMI could modulate plasma levels of miR-1, miR-133a/b and miR-499-5p in humans and mice [16]. A significant association of miR-195 and miR-30a with cardiac hypertrophy was reported using an in vivo mice model and mice cardiomyocytes [18,19]. To investigate the diagnostic potential, several cardiac origin miRNAs have been worked upon in blood, plasma, serum, and PBMCs in patients with stable and unstable angina (UA) [20]. Circulatory miRNAs are routinely found in systemic circulation with remarkable stability and in some pathophysiological conditions they can reflect tissue damage and often show tissue or “disease- specific” expression [21,22]. Moreover, the collection and processing of circulatory fluids is easily achievable, which has thus advanced the strategy of their application as pathological biomarkers in various disease conditions including myocardial injury [23,24]. 

The hunt for circulatory biomarkers in the form of miRNAs has thus become an integral part of investigations in cardiovascular research due to their potential to enable early detection of the disease [24]. Early diagnosis and prediction is crucial and can allow clinicians to risk stratify their patients in order to decide and select an appropriate course of treatment, and thus will have advantages in achieving relief from any sign and symptoms of coronary complications as well as in reducing the frequency of CAD incidences [25]. In the present study, we assessed the diagnostic property of circulatory miR-133b and miR-21 for early prediction and to distinguish CAD and its subcategories from healthy subjects. To the best of our knowledge, this is the first report demonstrating the differential expression profile of circulatory miR-133b and miR-21 in clinical as well as angiographically proven categories of CAD in a reasonable number of study subjects. 

## 2. Materials and Methods

### 2.1. Study Subjects

Ninety-three consecutive subjects, aged 35–80 years, admitted to Cardiology ward, all India Institute of Medical Sciences (AIIMS), a tertiary care center, New Delhi, who underwent coronary angiography (CAG) for suspected case of coronary atherosclerosis, were enrolled in this case-control study. Out of these, seventy-eight patients were clinically suspected and angiographically proven cases of CAD with reasonable stenosis (>75%) and followed inclusive criteria. Fifteen subjects were suspected cases but angiographically declared as NCA patients. These subjects had stenosis levels between 50% to 69% and were hence labelled as patients with pre-atherosclerotic condition. Fifty-four healthy participants of similar age group, who were attendants of the patients and were clinically unsuspected as they had no signs or symptoms of CAD, were enrolled in this study as control subjects. Based on earlier literature available on similar types of studies [14,16,17,20,22,25,26], we arrived at a decision of having an appropriate study size of about 70–80 each of cases and control. Now in order to get about 70 proven CAD cases, approximately 100 angiography cases had to be included. This sample size was expected to have an absolute error margin of 10% in a two-sided 95% confidence interval (CI) for an assumed sensitivity and specificity of >75% in detecting CAD.

Coronary angiography was performed for all the enrolled case subjects by cannulating the coronary arteries according to Judkins, Trans-radial or Transfemoral techniques, equipment, and practices standard to AIIMS cardiac catheterization laboratory. The diagnoses of CAD as well as NCA were made and confirmed by coronary angiogram and according to the American College of Cardiology (ACC)/American Heart Association (AHA) 2007 guidelines. Accordingly, subjects having > 70% luminal narrowing in at least one of the major epicardial coronary arteries were classified as CAD patients. Subjects with < 70% stenosis in any of the major coronary artery were classified as NCA subjects. Patients were classified according to the evidence from angiography report and clinical evaluations for chest discomfort. Chest discomfort is defined according to signs and symptoms such as uncomfortable pressure, tightness or heaviness, chest pain that has radiated from chest to neck, back, shoulder, jaw or one or both the arms with relentless dyspnea called uncomfortable or shortness of breath due to exertion. CAD patients were further categorized based on clinical tests and symptoms as SA and ACS, which further includes UA or NSTEMI and STEMI based on standard ACC/AHA definitions and guidelines. CAD patients were also categorized based on angiography results as single vessel disease (SVD), double vessel disease (DVD) and triple vessel disease (TVD) with reasonable stenosis in the major epicardial coronary arteries. Patients with more than 70% of stenosis, systolic blood pressure (SBP) of more than 140 mmHg, diastolic blood pressure (DBP) of 90 mmHg and diagnosed with diabetes mellitus (DM) and hypertension were included in the study. Patients with less than 50% stenosis while having any sort of valvular disease, cardiomyopathy, severe hepatic or renal dysfunction were excluded from the main study. All the study subjects had been residents of Delhi and/or surrounding areas for the previous three decades and their lifestyle and food habits were recorded as standard set of questionnaires. Clinico-pathological history of the patients and medication records were also procured from the record section, AIIMS, New Delhi.

### 2.2. Ethics Statements

The protocols adopted in the current study were conducted according to the ethical principles of the Declaration of Helsinki and its later amendments or comparable ethical standards. Certificates of application on the present proposal in the format of Indian Council of Medical Research (ICMR) were obtained and the study protocol was approved by Human Ethics committee, AIIMS, New Delhi and Human Ethics committee, ACBR, DU, Delhi. Human Ethics committee, AIIMS, New Delhi IEC/NP-207/2012 & RP-17/2012. Human Ethics committee, ACBR, DU, Delhi (F.50-2/Eth.Com/ACBR/169)2014. A written and signed informed consent form was obtained from all the participants of this study.

### 2.3. Baseline Clinical Characteristics of Study Subjects

The baseline clinical and laboratory characteristics of all the study subjects, selected for miRNA gene expression analysis, are listed in Table 1. All the CAD patients and healthy controls were of similar age group. Body mass index (BMI), obesity, SBP, DBP, DM, total cholesterol (TC), high-density lipoprotein cholesterol (HDL-C), low-density lipoprotein cholesterol (LDL-C), and hypertension records from the CAD patients were collected.

### 2.4. Collection of Blood Samples

Study samples were collected without revealing the identity of the subjects. All the researchers involved in handling and processing of the samples for total RNA isolation were blinded about the status of the study subjects to avoid any bias. Five milliliters of fasting venous blood was drawn in ice-cold EDTA containing tubes from the antecubital veins in the sitting position of all the study subjects in the morning before the cardiac catheterization procedure. To maintain the purity and integrity of the plasma, blood samples were handled with utmost care to avoid any potential disturbance and processed within 4 h of procurement of samples. Plasma was isolated from each of them using two-step centrifugation method. Blood samples were first centrifuged at 1200× *g* for 10 min at 4 °C to remove erythrocytes, and then supernatant was subjected to high-speed centrifugation at 16,000× *g* for 15 min at 4 °C in order to remove additional nucleic acids attached to cell debris and to obtain platelet-poor plasma. Finally, the plasma was transferred to RNase/DNase-free tubes for further processing.

### 2.5. Selection of miRNAs

Based on the previous studies [10,16,24,27], we selected miR-133b-5p and miR-21-5p as candidates for this study. MicroRNA-133b (miR-133b) is highly enriched in normal heart muscles. It is involved in many key aspects of normal heart development as well as complications including MI [10,24]. MicroRNA-21 (miR-21) was reported to be associated with UA in vulnerable CAD patients and also suggested to affect various aspects of plaque progression such as inflammation, extracellular matrix (ECM) degradation and angiogenesis [27]. 

### 2.6. RNA Isolation from Plasma Samples and Reverse Transcription

Total RNA enriched in RNA species smaller than 200 nucleotides, i.e., miRNAs were isolated from one milliliter of plasma samples using TRIzol LS reagent (Invitrogen, Thermo Fisher Scientific, Carlsbad, CA, USA) as per the manufacturer’s protocol with slight modifications. We increased the incubation time of upper aqueous phase with isopropanol at −20 °C from 10 min to overnight (~15 h). In addition, we added 2.5 μL of glycogen solution (10 mg/mL concentration) and sodium acetate (final concentration 0.3 M) in the upper aqueous phase to improve nucleic acid precipitation as plasma contains low amount of RNA. MicroRNA samples thus obtained were stored at −80 °C in RNase/DNase-free tubes until further processing.

### 2.7. Detection and Quantification of miRNAs by Quantitative Real-Time PCR

Each isolated RNA sample was polyadenylated and subjected to reverse transcription (RT) to prepare cDNA using the “TaqMan Advanced miRNA cDNA Synthesis Kit” (Thermo Fisher Scientific) as per the protocol provided by the manufacturer. cDNA was amplified using the miRNA specific “TaqMan^®^ Probes”, and “TaqMan Fast Advanced Master Mix” (Thermo Fisher Scientific) and detected using an ABI 7300 Real-time PCR system (Applied Biosystems^®^ Life Technologies) according to the manufacturer’s protocols for the corresponding microRNAs. Amplification was performed in triplicates in 20 μL PCR reaction, including 5 μL of cDNA, 1 μL of “TaqMan Advanced miRNA Assay” for each miRNA quantification. The relative expression level of miR-133b and miR-21 were calculated using comparative CT method. The analytical detection limit of miRNAs was defined and set as a CT value of 40; miRNAs with a CT value > 40 were considered undetectable, and thus those samples were excluded from the subsequent analysis. In all experiments, miR-186-5p was used as an endogenous control (TaqMan probe chemistry, ThermoFisher Scientific) to normalize the samples. The normalization process was performed by subtracting the CT values of miR-186-5p from CT values of both the miRNAs in patients as well as in control samples according to the formulae ∆CT = CT value of miR-133b or miR-21—CT value of miR-186-5p.

### 2.8. Statistical Analysis

Expression data of both the miRNAs were analyzed using Stata version 15.1 for Windows (Stata corp., college station, TX, USA), and presented as mean ± standard deviation. Quantitative variables were expressed as mean ± standard deviation and qualitative variables were expressed as proportions (%). A *p*-value < 0.05 was considered to be statistically significant. Normal Distribution of all the data sets were analyzed using Kolmogorov-Smirnov test, and they were found to be significant. Differences among the groups were compared using Student’s *t*-test. Qualitative variables were compared between the two groups using Chi-square test. The distribution of the miRNA expression in each group was presented as scatter/dot plot. One-way analysis of variance followed by Bonferroni correction for multiple comparisons was applied to assess the miRNA expression among controls and subcategories of CAD cases. Pearson’s correlation coefficient between study variables and miRNA expression in plasma were calculated along with the assessment for the significance of these correlations. The Receiver Operating Characteristic (ROC) curve analysis was done and the respective area under curve (AUC) was calculated in order to assess the predictive power of the circulatory miRNAs between CAD patients and healthy control group. The best threshold/cut-off value for each miRNA expression was identified and the sensitivity and specificity at that threshold value was determined to detect a CAD case, along with their 95% confidence intervals. Odds Ratios were calculated for study variables associated with CAD. A multivariable logistic regression analysis was carried out to assess the association of miRNAs expression with CAD, after adjusting for other study variables. Circulatory miRNAs expression data were used, and graphs were constructed using GraphPad prism version 8.1.1 (224) for Mac OS X (GraphPad software, San Diego, CA, USA) and reported as mean ± standard deviation.

## 3. Results

### 3.1. Clinical Characteristics of the Study Population with CAD and Healthy Subjects

A total of 78 CAD patients (49 males, 29 females), 15 NCA (8 males, 7 females) and 54 healthy volunteers (35 males, 19 females) of similar age were enrolled in the study. BMI, DM, hypertension, SBP, DBP, TC, HDL-C, LDL-C, and other treatment records were collected as shown in Table 1. The percentage of Hypertensive and DM incidences were higher and associated with miR-133b and miR-21 expression in CAD patients. Obesity and SBP also had a significant correlation with low miR-133b and high miR-21 expression level in CAD Patients.

### 3.2. Modulation in Plasma miR-133b and miR-21 Expression Level in CAD Patients

**Figure 1:**
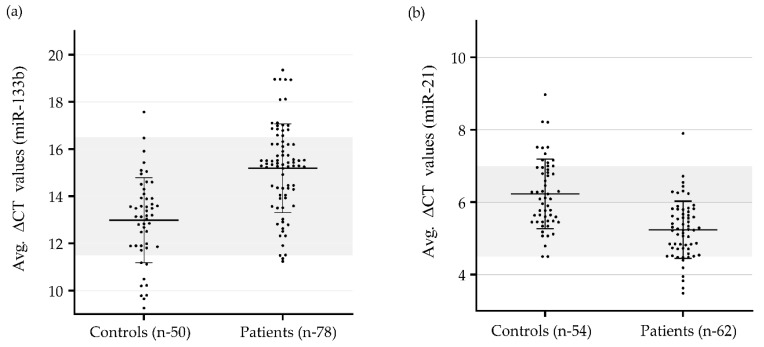

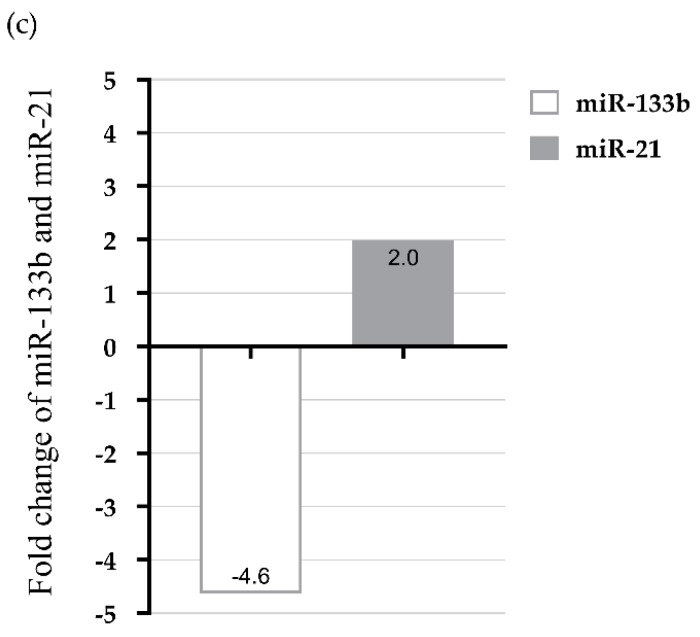
Quantitative real-time (qRT–PCR) analysis to find out variations in gene expression (delta-CT values) of miR-133b and miR-21 in the study subjects: (**a**) Differential gene expression (delta-CT values) of miR-133b in CAD patients (*n* = 78) vs. control subjects (*n* = 50). (**b**) Differential gene expression (delta-CT values) of miR-21 in CAD patients (*n* = 62) vs. control subjects (*n* = 54). Results were expressed as mean ± SD; (**c**) Relative fold changes of miR-133b and miR-21 in CAD patients compared to that of healthy subjects. MiR-21 was significantly upregulated, *p* < 0.0001 relative to control subjects. delta–delta-CT value of miR-21 for patients was found to be 1.99 (~2). MiR-133b was significantly downregulated, *p* < 0.0001 relative to control subjects. delta–delta-CT value of miR-133b for patients was found to be 2.21; Quantitative relative expression of miR-133b and miR-21 was calculated using comparative CT method (2^–ΔΔCT^) using *miR-186-5p* gene as an endogenous control to normalize the samples. Two-tailed Student‘s *t*-test was applied for comparison between the groups.

In our study, we found the average delta-CT value for miR-133b to be significantly higher (lower expression) in patients with CAD (15.19 ± 1.9, *p* < 0.0001) as compared to that in control group (12.98 ± 1.8, *p* < 0.0001). The delta–delta-CT value for patients was found to be 2.21. The average delta-CT value for miR-21 was significantly lower (higher expression) in patients (5.24 ± 0.79) as compared to that in the control group (6.23 ± 0.96). The delta–delta-CT value for patients was found to be −0.99. CT values of miR-133b ranged from 26.4 to 32.2 in patient samples and 21.9 to 30.8 in control samples. CT values of miR-21 ranged from 18.9 to 23.1 in patients and 18.8 to 24.3 in control subjects. No template control (NTC) samples were used in any of the experiments where NTC samples showed a value in the range between 38.0 and 39.5. The average delta-CT values of miR-133b and miR-21 of patient and control groups are shown in Figure 1a,b. The relative expression of miR-133b was found to be downregulated by 4.63 fold and that of miR-21 was upregulated by 1.99 (~2) fold in patients with CAD as compared to those of the control subjects (Figure 1c).

### 3.3. Expression Pattern of miR-133b and miR-21 in Different Clinical Categories of CAD

**Figure 2:**
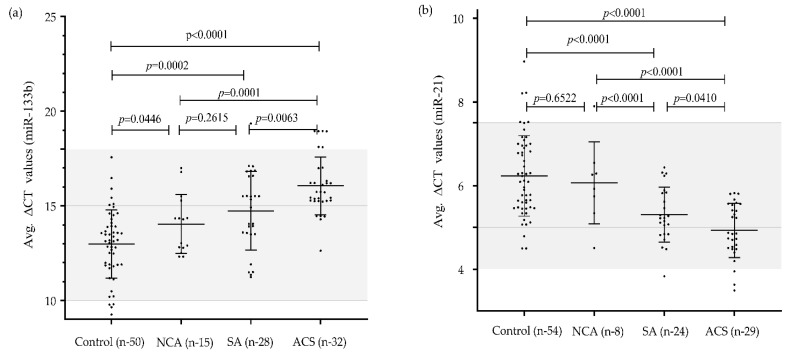
Mean expression values (delta-CT values) of miR-133b and miR-21 in different categories of CAD patients vs. control subjects: (**a**) Differential gene expression (delta-CT values) of miR-133b in different clinical categories of CAD Patients vs. Control subjects. A significant differential expression between control (12.98 ± 1.8; *n* = 50) and ACS (16.06 ± 1.5; *n* = 32); *p*-value < 0.0001, control vs. SA (14.74 ± 2.07; *n* = 28); *p*-value = 0.0002, control vs. NCA (14.05 ± 1.5; *n* = 15); *p*-value = 0.0446), NCA vs. ACS (*p*-value = 0.0001), SA vs. ACS (*p*-value = 0.0063) were obtained except NCA vs. SA (*p*-value = 0.2615); (**b**) Differential gene expression (delta-CT values) of miR-21 in different clinical categories of CAD patients vs. control subjects. A significant differential expression between control (6.23 ± 0.96; *n* = 54) and ACS (4.93 ± 0.64; *n* = 29); *p* value < 0.0001, control vs. SA (5.31 ± 0.65; *n* = 24); *p* value < 0.0001, NCA (6.06 ± 0.98; *n* = 8) vs. ACS (*p*-value < 0.0001), SA vs. ACS (*p*-value = 0.0410), NCA vs. SA (*p*-value < 0.0001) were obtained except control vs. NCA; *p*-value = 0.6522). Results were expressed as mean ± SD. One-way analysis of variance followed by Bonferroni correction for multiple comparisons was applied to assess the miRNA expression among controls and subcategories of CAD cases. *p*-value < 0.05 is considered to be significant.

**Figure 3:**
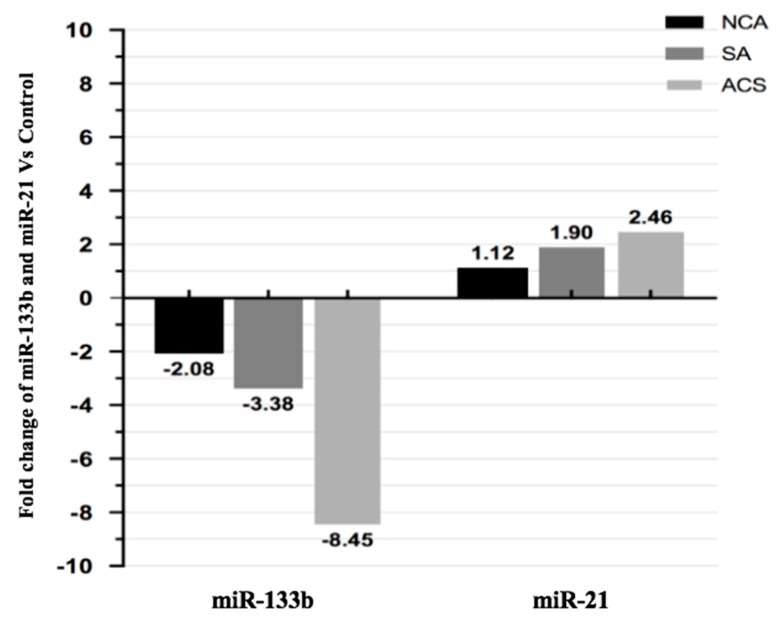
Relative fold changes of miR-133b and miR-21 in different categories of CAD compared to healthy controls. MiR-133b was downregulated by 8.45 fold in ACS, 3.38 fold in SA and 2.08 fold in NCA category patients as compared to healthy subjects. MiR-21 was upregulated by 2.46 fold in ACS, 1.90 fold in SA and 1.12 fold in NCA category patients as compared to healthy subjects.

We analyzed the expression levels of miR-133b and miR-21 in plasma of patients with SA, ACS and NCA to determine whether expression level of both the miRNAs were correlated with CAD or not. Figure 2 depicts the plasma concentration of both the miRNAs in different categories in scattered/ dot plot representation. The expression level of miR-133b was significantly downregulated (2.08-fold) in NCA (14.05 ± 1.5), 3.38-fold in SA (14.74 ± 2.07) and 8.45-fold in ACS (16.06 ± 1.5) as compared to that of the healthy subjects (12.98 ± 1.8). Similarly, miR-21 expression level was significantly upregulated by 2.46 fold in ACS subjects (4.93 ± 0.64) and 1.90 fold in SA (5.31 ± 0.65), but non-significantly, with a 1.12-fold change in NCA subjects (6.06 ± 0.98) as compared to healthy subjects (6.23 ± 0.96), Figure 3.

### 3.4. Association of Plasma miR-133b and miR-21 Levels with Clinical Categories within ACS Group

**Figure 4:**
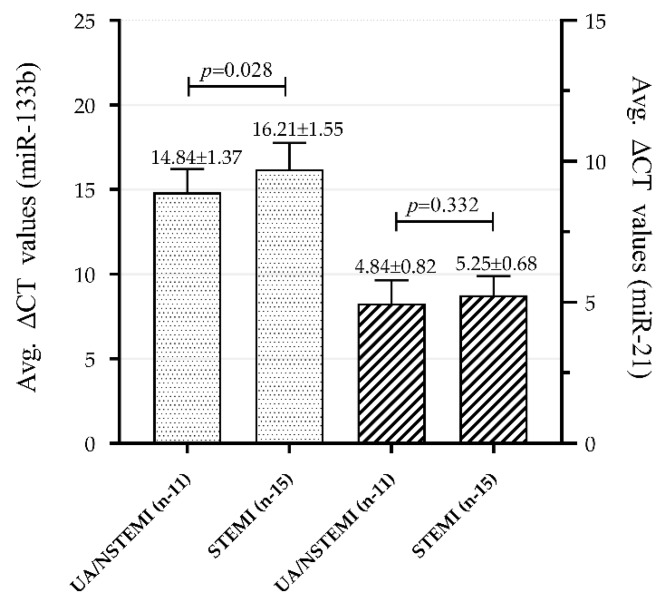
Mean expression values (delta-CT values) of miR-133b (left two bars) and miR-21 (right two bars) in subcategories of ACS in CAD patients. MiR-133b could significantly (*p* value = 0.028) differentiate UA/NSTEMI (14.84 ± 1.37; *n* = 11) category subjects from STEMI (16.21 ± 1.55; *n* = 15) subjects. MiR-21 could not show a significant (*p* = 0.332) difference between UA/NSTEMI (4.84 ± 0.82; *n* = 11) and STEMI (5.25 ± 0.68; *n* = 15) subjects. Data are means ± SD. Two-tailed Student‘s *t*-test was applied for comparison between the groups. *p* value < 0.05 is considered to be significant.

An altered expression pattern was observed in both the miRNAs in plasma samples of patients with subcategories of ACS group such as UA or NSTEMI (UA/NSTEMI) and STEMI, with STEMI being a more severe condition than NSTEMI. MiR-133b was able to significantly differentiate UA/NSTEMI (average ΔCT value of 14.84 ± 1.37) from STEMI (average ΔCT 16.21 ± 1.55) subjects, *p* = 0.028. Likewise, there was a clear expression difference in miR-21 between the two categories, average ΔCT 4.84 ± 0.82 vs. 5.25 ± 0.68 respectively; however, a significant difference was not observed (*p* = 0.332), as shown in Figure 4. These results indicate relatively lower expression of miR-133b and higher expression of miR-21 in STEMI as compared to NSTEMI/UA.

### 3.5. Association of miR-133b and miR-21 Expression Level with Angiography Result Based Categories and Severity of CAD

**Figure 5:**
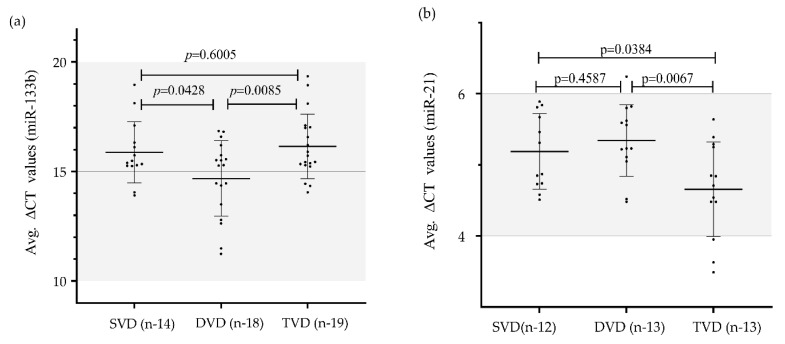
Mean expression values (delta-CT values) of miR-133b and miR-21 in different categories based on diseased vessels involved in CAD patients: (**a**) Differential gene expression (delta-CT values) of miR-133b in different categories of CAD based on diseased vessels involved vs. control subjects. Lowest expression of miR-133b was obtained in TVD (16.15 ± 1.47; *n* = 19) as compared to DVD (14.68 ± 1.72; *n* = 18) and SVD (15.88 ± 1.39; *n* =14). TVD vs. DVD (*p* value = 0.0085) and DVD vs. SVD (*p*-value = 0.0428) showed a significant difference for miR-133b except SVD vs. TVD (*p*-value = 0.6005); (**b**) Differential gene expression (delta-CT values) of miR-21 in different categories of CAD based on diseased vessels involved vs. control subjects. Results are expressed as mean ± SD. Highest expression of miR-21 was obtained in TVD (4.66 ± 0.67; *n* = 13) as compared to DVD (5.27 ± 0.44; *n* = 13) and SVD (5.34 ± 0.63; *n* = 12). TVD vs. DVD (*p*-value = 0.0067) and TVD vs. SVD (*p*-value = 0.0384) showed a significant difference for miR-133b except SVD vs. DVD (*p*-value = 0.4587). Results were expressed as mean ± SD. One-way analysis of variance followed by Bonferroni correction for multiple comparisons was applied to assess the miRNA expressions among the subcategories. *p*-value < 0.05 is considered to be significant.

To evaluate the relationship of plasma miR-133b and miR-21 with CAD, we further performed a sub-analysis to determine the association of these two miRNAs to the CAD severity based on angiographically proven categories as shown in Figure 5. CAD severity was assessed with the number of diseased vessels involved. The expression level of miR-133b was significantly lower in patients with TVD (16.15 ± 1.47) as compared to those with DVD (14.68 ± 1.72); *p* value = 0.0085 and SVD (15.88 ± 1.39); *p* value = 0.6005, where it could not reach a significant level. This suggests a negative correlation between levels of plasma miR-133b and severity of the disease. MiR-133b expression level was also significantly altered between DVD and TVD subjects (*p* value = 0.0428). Similarly, miR-21 expression was significantly higher in TVD (4.66 ± 0.67) than those of single vessel (5.34 ± 0.63); *p* value = 0.0384 or DVD (5.27 ± 0.44); *p* value = 0.0067. Thus, this indicates a positive correlation in the expression level of miR-21 with severity level of the disease. However, no significant difference was observed in miR-21 expression level between SVD and DVD (*p* value = 0.4587).

### 3.6. Expression Profile of miR-133b and miR-21 in CAD Patients with and without Diabetes Mellitus

We examined the expression profile of miR-133b and miR-21 both in CAD patients with DM and without DM. MiR-133b was observed to be downregulated by 3.91 fold in CAD patients with DM (*n* = 22) and downregulated by 4.93 fold in CAD patients without DM (*n* = 56). Similarly, miR-21 was observed to be upregulated by 1.82-fold in CAD patients with DM (*n* = 14) and upregulated by 2.04 fold in CAD patients without DM (*n* = 48).

### 3.7. Diagnostic Potential of miR-133b and miR-21

To further investigate the applicability and possibility of these circulatory miRNAs (miR-133b and miR-21) as novel and potential diagnostic biomarker of CAD, we performed area under the receiver-operating-characteristic (ROC) curve analyses. A relatively good predictive value/area under curve (AUC > 0.75) was obtained in CAD patients. 

**Figure 6:**
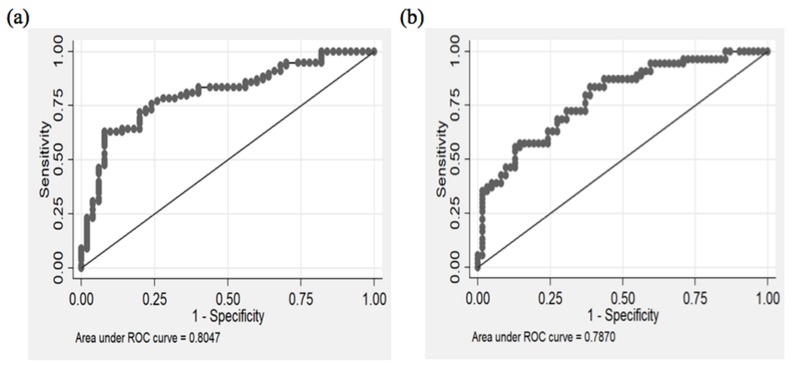
Diagnostic potential of circulating miR-133b and miR-21 through ROC curve: (**a**) Diagnostic potential of miR-133b, AUC = 0.80 (0.73 to 0.88 at 95% CI) with 75.6% sensitivity and 76% specificity; (**b**) Diagnostic potential of miR-21, AUC = 0.79 (0.70–0.87 at 95% CI) with 72.2% sensitivity and 69.4% specificity.

For instance, the ROC curve analysis (Figure 6a) showed that AUC for miR-133b was 0.80 (0.73 to 0.88 at 95% CI) with 75.6% (64.6 to 84.7 at 95% CI) sensitivity and 76% (61.8 to 86.9 at 95% CI) specificity when the relative expression level of miR-133b was at the recommended cut-off value of 14.0 (optimal cut-off point). This suggests that miR-133b exhibit a strong predictive power (80%) to distinguish CAD subjects from the healthy subjects. Subjects with the expression of miR-133b more than 14.0 were employed as subjects with susceptibility miRNA and susceptible to CAD. When the expression value of miR-133b was more than 14.0, the odds of having the disease was 9.83 (4.32 to 22.4 at 95% CI).

Likewise, the AUC value for miR-21 (Figure 6b) was 0.79 (0.70–0.87 at 95% CI) with 72.2% sensitivity and 69.4% specificity at the recommended optimal cut-off value of 5.59, which exhibited a good predictive power (79%) in distinguishing CAD patients from control subjects. Subjects with miR-21 expression values less than the optimal cut-off value of 5.59 were employed as subjects susceptible to CAD where the odds of having the disease were 4.93 (2.25 to 10.8 at 95% CI). Further details of this analysis for the miRNAs have been provided in Table 2. Therefore, our results suggest that miR-133b and miR-21 in plasma might be helpful and can be used as potential diagnostic biomarkers for the identification and prediction of CAD patients.

### 3.8. Diagnostic Potential of miR-133b for Different Categories of CAD

**Figure 7:**
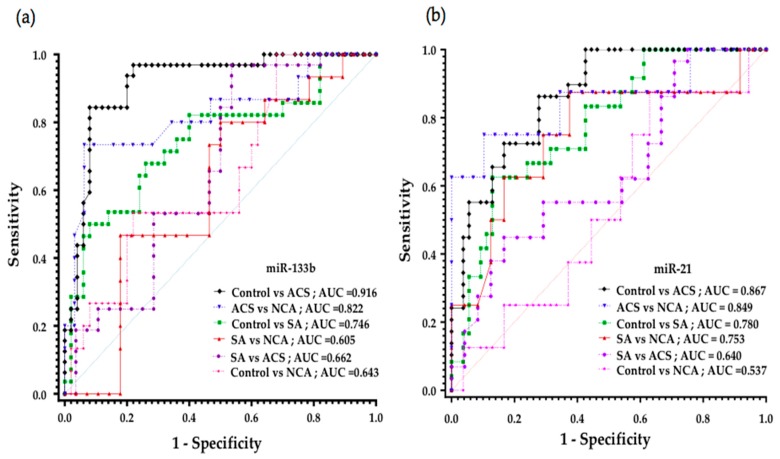
Diagnostic potential of circulating miR-133b and miR-21 as analyzed by ROC curve in different clinical categories of CAD: (**a**) ROC curve of miR-133b in different categories of CAD. Diagnostic potential of miR-133b as represented through AUC values was 0.916 for ACS, 0.746 for SA and 0.643 for NCA category as compared to control subjects; (**b**) ROC curve of miR-21 in different categories of CAD. Diagnostic potential of miR-21 was 0.867 for ACS, 0.780 for SA and 0.537 for NCA category as compared to control subjects.

ROC analysis was carried out to evaluate the discriminatory power of miR-133b for various categories or events of CAD such as SA and ACS in comparison to control subjects as well as pre-atherosclerotic condition such as NCA subjects, Figure 7a. A significant altered expression of miR-133b (delta-CT value) was observed between ACS and SA when compared to healthy subjects, *p* ≤ 0.0001 and 0.0002, respectively. This was evident from the area under ROC curve (AUC) results; ROC = 0.916 (0.85–0.98, at 95% CI, *p* < 0.0001) for ACS vs. healthy subjects, 0.7464 (0.62–0.87, at 95% CI, *p* = 0.0003) for SA vs. healthy subjects, thus showing the diagnostic potential of miR-133b to accurately discriminate ACS and SA subjects from healthy subjects.

miR-133b expression was also significantly altered between ACS subjects as compared to pre-atherosclerotic NCA disease subjects (*p* = 0.0001) and thus can discriminate the ACS category from NCA. Interestingly, miR-133b could significantly differentiate pre-atherosclerotic NCA disease subjects from healthy individuals (*p* = 0.0446). This gives the idea that miR-133b gives a potent advantage to predict the disease condition in pre-atherosclerotic stage itself prior to severe and late-stage conditions such as ACS. MiR-133b could also significantly discriminate between ACS and SA subjects (*p* = 0.0063). However, in our study, a significant difference in miR-133b expression was not observed between SA and NCA (*p* = 0.2615).

### 3.9. Diagnostic Potential of miR-21 for Different Categories of CAD

There was a significant difference in miR-21 expression between ACS and healthy individuals (*p* ≤ 0.0001), as well as between SA and healthy individuals (*p* ≤ 0.0001), which was also evident from the area under ROC curve (AUC) results; ROC = 0.867 (0.7913 to 0.9417 at 95% CI, *p* <0.0001) for ACS vs. healthy subjects and 0.7801 (0.67–0.89, at 95% CI, *p* < 0.0001) for SA vs. healthy subjects, thus showing its diagnostic potential to discriminate ACS and SA from healthy subjects Figure 7b. 

A significant altered expression of miR-21 was observed in ACS as compared to NCA (*p* = 0.0004) as well as SA subjects as compared to NCA (*p* = 0.0182,), and thus suggests that miR-21 can discriminate between ACS and NCA subjects as well as between SA and NCA. Likewise, miR-21 could also significantly discriminate ACS from SA category (*p* = 0.0410). However, a significant difference in miR-21 expression was not observed between NCA and healthy individuals (*p* = 0.6522).

### 3.10. Interaction of Plasma miR-133b and miR-21 with Classical Risk Factors in CAD Patients

Pearson correlation analysis was performed for positive or negative association between miR-133b, miR-21 and classical risk factors. Both the miRNAs showed negative correlation to each other, *p* = 0.007, which is also evident from the expression results. SBP was negatively correlated with miR-21 (*p*-value = 0.03) but positively correlated with 133b (*p*-value = 0.03). LDL and HDL showed positive correlation to miR-21 with no statistical significance (*p*-values 0.39 and 0.96, respectively) and negative correlation to miR-133b with no statistical significance (*p*-values = 0.78, 0.46, respectively).

### 3.11. Regression Model for miR-133b and miR-21

As shown in Table 1, most of the clinical features did not show any significant difference between CAD patients and control subjects. Exceptions were age, obesity, and SBP (*p* < 0.05). Multivariable logistic regression analysis revealed a significant association of both the miRNAs with CAD even after adjusting for obesity and age as confounding variables. MiR-133b (Adjusted odds ratio (OR) 11.91 (3.90–33.96) at 95% CI, *p* < 0.05), miR-21 (Adjusted OR 6.83 (2.31–20.22) at 95% CI, *p* = 0.001), Obesity (Adjusted OR 6.97 (1.86–26.19) at 95% CI, *p* = 0.004) and age (Adjusted OR 1.05 (0.99–1.11) at 95% CI, *p* = 0.93) were involved in the regression model. This suggests that both the miRNAs are independently associated with the risk of CAD.

## 4. Discussion

Despite tremendous advancements in treatment therapies and care, CAD is still a major cause of heart attacks and contributor in mortality rates globally [1,28]. MicroRNAs have emerged as stable blood-based biomarkers in numerous diseases including CAD [7,21]. Based on their tissue selectivity, several “cardiomyocyte-enriched” miRNAs have been investigated for their potential as diagnostic biomarkers and their association with AMI [16,24]. Their quick release into the circulation from the damaged cells due to rapid release dynamics under disease conditions makes miRNAs prominent and efficient candidates for diagnostic tools in the evaluation of many chronic conditions including CAD [26]. Circulatory miRNAs are highly resistant to degradation because of their small size and they are even sheltered from endogenous RNase activities as they are found to be linked with certain lipid-based carriers or encased within micro vesicles [29]. With such advantages over other conventional methods of diagnosis, circulatory miRNAs have emerged as a great and exciting opportunity with respect to the investigation of novel regulators of cardiovascular diseases including CAD. 

MicroRNAs, as endogenous molecules, have gained particular attention due to their significant role as micromanagers in gene regulation. Certain tissue- and “cell-specific” miRNAs have been clearly shown to be expressed in/and associated with cardiovascular settings and involve in maintaining vascular integrity [30]. Emerging evidence suggests that any changes or disruption into the arterial flow modulate the expression profiles of miRNAs of varied cells of luminal wall [31,32]. Studies have shown that several flow-sensitive miRNAs including miR-21, miR-23b, miR-92a, miR-19a, miR-143, miR-145, and miR-155, miR-205, miR-126 are directly or indirectly involved in the pathophysiological mechanism of atherosclerosis and therefore identified as mechano-miRs [31]. Interestingly, data from a large sequencing project on healthy adult heart identified several miRNAs such as miR-1, miR-16, miR-27b, miR-30d, miR-126, miR-143, miR-208 to have high expression in healthy cardiac tissue [33], thus suggesting that these miRNAs are likely to play a pivotal role in cardiac development as well as disease pathophysiology [6,34]. Moreover, the dissimilarity in the differential expression pattern of circulatory miRNAs among different population has been demonstrated by numerous studies, suggesting that ethnicity might also play a key regulatory role [35,36]. In addition, even prognostic values of miRNAs have been shown to be influenced by the patient’s race/ethnicity [37].

The role of circulatory miR-133b and miR-21 in plasma samples of CAD subjects is unknown and scarcely studied. In the present study, we have demonstrated the association of altered expression profile of circulatory miR-133b and miR-21 in patients with CAD in comparison with healthy individuals. We also tried to demonstrate the potential of both the miRNAs to discriminate atherosclerotic condition (CAD) from pre-atherosclerotic condition (NCA). MicroRNA-133b (miR-133b) has been predominantly appraised as a “muscle-specific” miRNA [38], and thus termed as myomiR. It is coded by the gene MIR133b on chromosome 6 and transcribed as a bicistronic transcript with miR-206 as miR-206/miR-133b cluster. MicroRNA-133b (miR-133b) is among the most abundant muscles or/and “cardiac-specific” miRNAs present in the normal heart. It is involved in heart development as well as diseases related to it including MI [16,39] and cardiac hypertrophy [40], where miR-133b was shown to have an opposing effect. MiR-21, on the other hand, has previously been studied for its role in the pathogenesis of MI, ischemia/reperfusion injury and thus identified to have its multifunctional role in cardiovascular system [41,42]. These studies demonstrated the contrasting character of miR-21, and thus indicated that the role of miR-21 in cardiovascular settings is context dependent. Recently, researchers also tried to identify the role of miR-21 in AMI patients [43] but also addressed the limitation of its “cardiac specificity”, as miR-21 is involved in multiple organs. This issue can certainly be overcome by using it in combination with other miRNA signatures as diagnostic markers as shown by other studies [32,44]. 

In light of the above studies, wherein, using animal models, tissue samples and “cardiac-specific” cell lines, the utility of various circulatory miRNAs was shown to have significant diagnostic potential for cardiovascular complications, we decided to ascertain the potential role of miR-133b and miR-21 as non-invasive diagnostic biomarkers in CAD. Previously, Ai J et al. (2010) showed miR-133b expression in 93 AMI patients’ samples, but the results were not statistically significant [45]. Similar observations were reported by Widera C et al. (2011) [26]. In contrast, Liu H et al. (2019) showed a negative correlation of miR-133b with atherosclerosis in a mice model [46], whereas Bostjancic E et al. (2010) found similar results but a significant expression difference could not be observed in autopsy samples of MI patients [39]. Wang E et al. (2013) showed lower expression in 7 MI patient samples who underwent heart transplant [47]. Cortez-Dias et al. (2017) reported the prognostic potential of miR-133a/b by using serum samples of STEMI patients who underwent a primary angioplasty [48]. Other studies showed contrasting results for miR-133b [16,49,50]. Thus, conflicting reports on the role of miR-133b in different cardiovascular diseases necessitated further investigation in this direction. Hence, the potential contribution of these miRNAs in the disease pathophysiology is no doubt an important aspect to be explored.

Using 78 CAD patients, our study provides evidence for the significant association of miR-133b (negative correlation), and miR-21 (positive correlation) to the risk of CAD. We found that miR-133b expression in plasma samples of CAD patients was markedly downregulated by 4.63 fold, whereas miR-21 expression was upregulated by ~2 fold as compared to healthy individuals. The results for miR-133b expression pattern were in line with the observations of recently reported studies [39,45,46,47]. We also examined the expression profile of both the miRNAs in CAD patients with and without DM as compared to healthy subjects. Interestingly, CAD patients with and without DM did not show any significant deflection in the fold change of miR-133b and miR-21 expression as compared to the fold change observed in overall CAD patients compared to healthy individuals. This suggests that the presence or absence of DM in the CAD subjects of our study did not have any significant effect on the expression pattern of both the miRNAs. It is important to mention that the development of CAD is a result of several chronological events such as endothelial activation and its dysfunction, plaque formation and rupture, aggregation of blood cells including platelets and necrotic instability of myocardium [27,51]. Hence, it is evident that numerous miRNAs, derived from different cell types which are associated with a specific stage of the disease, might show their differential expression pattern at any time during the course of the disease.

In the present study, we also found that the expression profile of both the miRNAs starts changing before the actual severity level of the disease comes into the process. Based on our results, the expression pattern of both the miR-133b and miR-21 starts changing even in NCA subjects, who are referred to as pre-atherosclerotic subjects, wherein the extent of stenosis can range from 50% to 69%. NCA was considered to be a pre-atherosclerotic stage where the subjects are otherwise considered to be angiographically proven normal subjects with no significant stenosis in any of the major coronary arteries. MicroRNA-133b (miR-133b) could significantly (*p* = 0.0446) differentiate NCA condition from healthy subjects, with a fold difference of 2.08. This suggests that even when the NCA condition is considered or close to the healthy stage, there were significant differences in the expression of miR-133b in NCA subjects as compared to healthy subjects, possibly due to the extent of stenosis (50%–69%). These results may suggest that miR-133b can predict pre-atherosclerotic condition from rest of the stages of CAD. MicroRNA-21 (miR-21) also showed a clear difference between healthy subjects and the pre-atherosclerotic NCA condition, with a fold difference of 1.12. In conclusion, pre-atherosclerotic subjects showed similar trends of expression of both the miRNAs as shown in patients with higher stenosis. These results should be further confirmed using a large data set, but this surely provides an indication that miR-133b and miR-21 have potential to differentiate an early stage or pre-atherosclerotic stage (NCA) from CAD comprising the SA and ACS condition. 

Our results suggest a strong association of plasma miR-133b and miR-21 expression with atherosclerotic disease severity. MiR-133b showed a trend of gradual decline in its expression level as the disease progress from pre-atherosclerotic condition towards its more severe clinically progressive form, such as ACS, where it showed its lowest expression. Similarly, miR-21 showed an upregulation in its expression as the disease progresses from NCA to ACS. This suggests that both the miRNAs have the potential to discriminate ACS from other categories of CAD compared to healthy subjects, and thus can serve as potential diagnostic biomarkers of development and progression of CAD in order to predict disease complications at an early stage before it gets worse.

Our hypothesis was further substantiated by the observation that the lowest expression level of miR-133b was observed in TVD (most severe) condition (negative correlation) compared to higher expression in DVD and SVD. Likewise, the highest expression level of miR-21 was observed in TVD (positive correlation) as compared to that in subject with SVD and DVD conditions. 

To further elucidate the association of miR-133b and miR-21 with ACS, we attempted to investigate the potential role of both the signatures in discriminating different pathological conditions and categories within ACS, i.e., STEMI and UA/NSTEMI. We observed a significantly lower expression pattern of miR-133b in most severe STEMI condition as compared to the other category (UA/NSTEMI), thus indicating its potential to differentiate between the two categories. In our study, both the categories showed an altered expression of miR-21, but a significant difference was not observed. Until now, very few researchers have studied the role of miR-133b and miR-21 in the ACS condition specifically in its subcategories (STEMI and UA/NSTEMI). 

Diagnostic potential to identify the discriminatory/predictive power of miR-133b and miR-21 was assessed by means of area under ROC curve analysis. MiR-133b and miR-21 were found to be highly predictive as potential biomarkers of CAD. Diagnostic potential and predictive power of miR-133b and miR-21 with respect to CAD was as high as 80% and 79% respectively, with an upstanding sensitivity and specificity. Moreover, ROC results showed that both the miRNAs could predict ACS condition more prominently and precisely compared to SA and other conditions, thus suggesting their high utility as non-invasive diagnostic biomarkers in the CAD condition.

The current study provides the first clinical evidence of circulating miR-133b and miR-21 as novel non-invasive predictive biomarker in diagnosing CAD in Indian subjects. Our study consists of study subjects from the north India region and surrounding areas. As this is a single/limited region study, a multiregional or multicentric approach may provide a broad picture which can further enhance the validity of the prospective results. So far, not many efforts have been directed towards establishing a significant association between some of the circulatory candidate miRNAs and the severity of CAD when developing a non-invasive diagnostic method that can help in reducing coronary complications. Considering CAD as a multifactorial and multigenic disease driven by various genetic and non-genetic factors, observations from the current study appear to support the assumption that miRNA signatures have important implications on CAD and its different stages.

## 5. Conclusions

Based on the results from the current study, miR-133b and miR-21 are significantly associated with the risk of CAD and different subcategories. Both the miRNAs have a potent ability to discriminate and predict various stages of the disease from pre-atherosclerotic to its severe atherosclerotic form. Observations from the current study, although exploratory, shed some light on the pathophysiological role of miRNAs, thus propounding their potential inclusion in coronary artery complications. A large cohort of patient studies are still warranted in order to unequivocally establish the potential diagnostic utility of miR-133b and miR-21 in rapid diagnosis of CAD patients and their subcategories, where conventional biomarkers, such as cardiac troponins are less reliable or are of invasive nature. In conclusion, together with the previous reports and the findings from the current study, the advantages provided by miRNA signature shows their potential to be utilized as biomarkers of early prediction of coronary complications in patients with CAD, thus indicating their integration with other conventional parameters such as cardiac troponin in order to improve the diagnosis of vascular diseases such as CAD.

## Figures and Tables

**Table 1:** Baseline clinical characteristics of all the study subjects.

Parameters	Controls (*n* = 54)	Patients with CAD (*n* = 78)	Patients with NCA (*n* = 15)	*p*-Value ^†^	*p*-Value ^‡^
Age (years)	53.1 ± 11.5	58.7 ± 9.4	60.4 ± 9.6	0.0028 *	0.0286 *
Gender (Male %)	64.8	62.8	53.3	0.8166	0.4244
SBP (mmHg)	122.7 ± 8.4	132.9 ± 17.7	130.9 ± 15.4	0.0003 *	0.0112 *
DBP (mmHg)	79.5 ± 6.3	80.3 ± 14.2	80.33 ± 22.3	0.7192	0.8234
BMI (Kg/m^2^)	24.87 ± 5.4	25.76 ± 3.2	24.22 ± 5.7	0.2804	0.1879
Obesity (%)	12.96	42.3	46.7	0.0002 *	0.0036 *
Diabetes mellitus (%)	0	28.2	26.7	-	-
Hypertension (%)	0	57.7	53.3	-	-
LDL-C (mg/dL)	102.4 ± 34.7	93.15 ± 24.9	89.56 ± 20.55	0.1029	0.2871
HDL-C (mg/dL)	41.54 ± 13.3	41.91 ± 11.1	44.67 ± 10.55	0.8727	0.5076
TC (mg/dL)	177.74 ± 42.03	176.76 ± 34.11	157.47 ± 52.45	0.8908	0.0964

Data are means ± SD and mean percent values. Mean values of the different parameters in the study subjects were analyzed by chi-square and Student’s *t*-test of significance as appropriate; *p*-value **^†^**—Patients with CAD vs. Controls. *p*-value **^‡^**—Patients with NCA vs. Controls. * *p* < 0.05 is considered to be significant. Age (*p*-value—0.0028 **^†^** and 0.0286 **^‡^**), SBP (*p*-value—0.0003 **^†^** and 0.0112 **^‡^**) and Obesity (*p*-value—0.0002 ^†^ and 0.0036 ^‡^) were found to be significantly different between the groups.

**Table 2:** Receiver operating characteristic curve (ROC) analysis of miR-133b and miR-21 for their diagnostic potential for coronary artery disease.

	AUC (95% CI)	Cut pt.	Odds Ratio (95% CI)	Sensitivity (95% CI)	Specificity (95% CI)
**miR-133b**	0.80 (0.73–0.88)	14.0	9.83 (4.32–22.4)	75.6% (64.6–84.7)	76% (61.8–86.9)
**miR-21**	0.79 (0.70–0.87)	5.59	4.93 (2.25–10.8)	69.4% (56.3–80.4)	72.2% (58.4–82.5)

ROC curve analysis of miR-133b and miR-21 showed strong discrimination between CAD and control subjects. MiR-133b showed AUC value—0.80 (0.73 to 0.88 at 95% CI) with 75.6% sensitivity and 76% specificity at the recommended cut-off point of 14.0. MiR-21 showed AUC value—0.79 (0.79 to 0.87 at 95% CI) with 69.4% sensitivity and 72.2% specificity. Cut pt.: optimal cut-point is determined by largest sum of specificity and sensitivity values.
